# Voice and Handgrip Strength Predict Reproductive Success in a Group of Indigenous African Females

**DOI:** 10.1371/journal.pone.0041811

**Published:** 2012-08-03

**Authors:** Jeremy Atkinson, R. Nathan Pipitone, Agnieszka Sorokowska, Piotr Sorokowski, Mara Mberira, Astrid Bartels, Gordon G. Gallup

**Affiliations:** 1 Department of Psychology, University at Albany, State University of New York, Albany, New York, United States of America; 2 Department of Psychology, Adams State University, Alamosa, Colorado, United States of America; 3 Institute of Psychology, University of Wroclaw, Poland; 4 Faculty of Humanities and Social Science, University of Namibia, Windhoek, Namibia; 5 Technische Universitaet Berlin, Institut für Sprache und Kommunikation, Berlin, Germany; University of Utah, United States of America

## Abstract

Evolutionary accounts of human traits are often based on proxies for genetic fitness (e.g., number of sex partners, facial attractiveness). Instead of using proxies, actual differences in reproductive success is a more direct measure of Darwinian fitness. Certain voice acoustics such as fundamental frequency and measures of health such as handgrip strength correlate with proxies of fitness, yet there are few studies showing the relation of these traits to reproduction. Here, we explore whether the fundamental frequency of the voice and handgrip strength account for differences in actual reproduction among a population of natural fertility humans. Our results show that both fundamental frequency and handgrip strength predict several measures of reproductive success among a group of indigenous Namibian females, particularly amongst the elderly, with weight also predicting reproductive outcomes among males. These findings demonstrate that both hormonally regulated and phenotypic quality markers can be used as measures of Darwinian fitness among humans living under conditions that resemble the evolutionary environment of *Homo sapiens*. We also argue that these findings provide support for the Grandmother Hypothesis.

## Introduction

Growing evidence shows that many sexually dimorphic traits in humans convey adaptive (evolutionarily relevant) information. The voice seems to be one medium that provides such biological information. Individuals with attractive voices tend to have more attractive bodies [1], increased mating success [2], and show fewer deviations from bilateral symmetry [3]. Fundamental frequency (of which voice pitch is the acoustic percept) is a vocal parameter that correlates with perceptions of attractiveness and mate quality. Males with voices lower in fundamental frequency are rated by females as more attractive, dominant and masculine [4]–[9]. Females with voices higher in fundamental frequency (based on natural differences as well as experimental manipulation) are rated as more attractive [10]–[13] and have other characteristics that confer femininity [14]. Differences in vocal parameters are thought to be due to differences in testosterone exposure [15]–[18] for both males and females, and estrogen exposure in females [6], [19]. The degree of sexual dimorphism in human vocal traits as a consequence of testosterone exposure signals phenotypic quality [5], [14], [20] and reported mating success [21] in males. Among females, reproductive viability [5], [10], [19], reproductive development and health are also related to estrogen exposure [22], [23]. Recent reviews [24], [25] have summarized literature showing that the mere sound of a person’s voice conveys information about the speaker’s gender, age, body configuration, hormonal status, sexual behavior, strength, fertility, as well as subtle deviations from bilateral symmetry – all of which bear on health and fitness. Thus, differences in vocal traits among males and females appear to reflect real biological differences in reproductive potential.

Only one study to date has shown correlations between vocal acoustics and reproductive success (RS) in an environment resembling human evolutionary history; Apicella et al. [26] reported that male fundamental frequency predicted the number of living children ostensibly sired by male Hadza, an indigenous hunter-gatherer population living in Tanzania. However, paternity tests were not used, and complete assurance of paternity is therefore suspect. As the authors themselves acknowledge, the results could be due to increased paternal confidence, not necessarily increased RS. Among females, maternity is almost always certain, outside of maternity ward confusion in Western society. Thus, we hypothesize that males with a lower fundamental frequency and females with a higher fundamental frequency will have increased reproductive output within the present sample.

Handgrip strength (HGS) is both a sexually dimorphic trait and a robust marker of phenotypic quality, health, and an indirect measure of fitness [27]. Handgrip strength is related to masculine features and increased androgen exposure in males and is correlated with organizational (i.e., prenatal) effects of testosterone [28], as well as activational effects of testosterone in elderly [29], normal [30] and hypogonadal/low serum testosterone men [31]–[34]. Increases in HGS correlate with testosterone increases in pre-adolescent athletes [35], and to waking testosterone levels in physically active men [36]. Handgrip strength is heritable [37]–[40], and is an informative measure of a male’s overall physical prowess, social aggression, body morphology and sexual behaviour [27], [41], [42]. Additionally, HGS is correlated with lean muscle mass [43] and male facial attractiveness [42]. Handgrip strength is a vital component of upper body strength, which is a component of perceived male strength from voice ratings [44] and perceived fighting ability [45] in both Western and indigenous populations. Thus, we hypothesize that higher handgrip strength, after controlling for variables such as height and weight, will be correlated with increased reproductive output among males.

However, in addition to a clear androgen and genetic component, HGS is also a robust predictor of phenotypic quality in both sexes throughout a person’s entire life. For example, large scale cohort studies have determined that adult HGS is correlated with higher birth weight in both sexes, independent of adult body height and weight [46], [47], suggesting that adult HGS is a good measure of the quality of the prenatal environment for both sexes. Additionally, numerous studies have found that HGS is correlated with general measures of health in adults [48], as well as current nutritional status in men and women of all ages [49]. HGS is also an excellent predictor of longevity and health in the elderly. Higher HGS is associated with reduced elderly mortality and disability [50]–[56], and low HGS in adults is highly predictive of later-life mortality and disability levels [49], [56]–[60]. Handgrip strength predicts hospitalized patients’ protein loss [61] as well as bone mineral density in healthy subjects [62], [63], post-menopausal and/or elderly women [64], [65] and adolescent girls [66]. Higher HGS is correlated with reduced risk of cognitive diseases [67] and muted cognitive decline in seniors [68]–[73]. Therefore it is hypothesized that, within each sex, higher HGS will predict increased RS in both sexes due to the large and robust literature linking HGS to phenotypic quality.

There is also a theoretical *a priori* reason why HGS could be positively correlated with increased RS in females. The Grandmother Hypothesis posits that the extended post-menopausal lifespan in human females is an adaptive trait that enables women to assist and provision their children’s children [74]–[77]. Traditional investigations of the Grandmother Hypothesis have focused on RS differences in mothers based on the presence or absence of their mother [e.g., 78]. Another way to investigate the Grandmother Hypothesis is to determine whether phenotypic quality markers directly relating to older women’s ability to provide resources for their progeny’s children positively correlates with their RS. Handgrip strength is an excellent candidate for such a marker, as much of the investment grandmothers provide involves taking over the “most arduous domestic tasks” [79] faced by mothers. These tasks include collecting firewood and/or gathering and preparing hard-to-acquire food, such as tubers [80]. This help enables mothers to spend more time in direct childcare and to forage for easy-access food with their children [80], which in turn decreases infant mortality [75] and increases growth rate [74], increasing the quantity and quality of her offspring [79]. In many traditional populations, older women (which include grandmothers) provide hard-to-obtain resources for their families [80], whereas returns from hunting dramatically decrease in older males [81], [82] due in part to their faster rate of senescence [83]. Recent research has shown that male HGS senesces faster and earlier than female HGS [84], suggesting that the maintenance of HGS might be more evolutionary important for elderly females. Thus, we hypothesize that HGS will predict RS among elderly females, but not elderly males.

In summary, the present study investigated the relationship between RS and both fundamental frequency and HGS in an indigenous population, with particular attention to females as maternal confidence is not suspect. We also use several measures of reproductive output to assess genetic fitness to a fuller extent. Additionally, we use handgrip strength as a marker of phenotypic quality, and its potential to relate to the Grandmother Hypothesis.

## Methods

Anthropometric, reproductive, and vocal samples were collected from the Himba (Ovahimba), who live in the northwest region of Namibia. The data were collected by the two Polish authors (P.S. and A.S.) and this research was approved by the Polish Ethical Committee at the University of Wroclaw, Poland. Our Namibian colleagues from The University of Namibia at Windhoek did not know of any law requiring ethical committee approval for a study involving anonymous, non-invasive data collection, therefore all ethical issues were handled by the University of Wroclaw. Because the population studied was illiterate, written consent could not be obtained, thus participants gave verbal consent and were told that their participation was voluntary and that they could quit at any time, without loss of monetary compensation. Obtaining verbal consent was also approved by the University of Wroclaw.

The Himba are one of the few ethnic groups in Africa who have preserved their traditional lifestyle, including wearing traditional clothing as well as maintaining most of the beliefs and customs of their ancestors [85]. They live a nomadic lifestyle contingent upon resources in and around the outskirts of Opuwo, an area rarely visited by tourists. All participants lived in small traditional villages, where only a few inhabitants (10 to 15 people, or 2 to 3 families) occupy each village. There is little contact with Western culture and no large-scale development. None of the villages we visited had access to contraceptives. Additionally, there is extremely limited access to formal education and modern medicine. However, a mobile school did make infrequent visits to some of the small villages. The Himba economy is based on livestock pasturing and supported by hunting, small-scale gardening and gathering [85]. Able-bodied Himba men spend the majority of time herding cattle and goats, while women perform most other tasks, including milking cows, constructing houses, fetching water from wells, and caring for children. All adults in the surrounding villages were invited to participate in the study. Participants were paid a small fee, equal to approximately $3 USD. Although the data were collected in one village, individuals who heard of the study and the monetary compensation came from neighbouring villages to participate. The final sample consisted of 54 females aged 18–80 (41.57±18.7) and 36 males aged 18–76 (50.36±18.1).

Generally speaking, we were less likely to encounter men in villages compared to women, as able-bodied men are usually herding. Women in the village confirmed our observations. Men that were available to participate were often elderly or physically weak (P.S., A.S., M.M., personal communication). Participating males were significantly older than participating females (*t_90_* = 2.34, *p* = .021). Age was self-estimated by all subjects and was often based on personal events. The majority (84%) of the group was married and 42% of men had more than one wife. A total of 101 individuals participated in the study, with six participants under the age of 18, three statistical outliers, and two participants who did not provide a voice sample and were removed prior to analyses. Descriptive statistics can be found in Table 1.

**Table 1:** Descriptive statistics of variables used in the study.

	children	grandchildren	genetic vectors	age	fundamental frequency (Hz)	height (cm)	weight (kg)	handgrip strength (bars)
males								
mean	4.83	2.13	5.9	50.36	145.48	174.44	65.83	6.04
s.d.	3.53	3.03	4.44	18.1	21.94	7.2	9.63	1.54
N = 36								
females								
mean	3.43	1.75	4.33	41.57	236.59	165.61	63.8	4.5
s.d.	2.34	3.09	3.25	18.7	38.17	5.2	10.58	1.41
N = 54								

Note: Genetic vectors composes of the total number of surviving children plus the number of grandchildren weighted by the presence of shared genes, or (number of children + (.5×grandchildren)).

The participants were interviewed by a citizen of Namibia fluent in the local dialect. They were asked questions regarding their marital and reproductive history. Subjects were asked to indicate their total number of children and grandchildren, and the number still alive. From this latter number, a novel measure of RS, *genetic vectors (GV),* was calculated as follows: 1*(number of living children) + ½ *(number of living grandchildren). The number of grandchildren was halved to calculate a measure of genetic fitness based on the proportion of shared genes. Genetic vectors take into account the reproductive avenues available to an individual through their children and grandchildren and provides a reasonable estimation of lifetime fitness for an indigenous population that is subject to high levels of mortality (particularly infant mortality), has overlapping reproduction among generations and could only be measured at one specific time by researchers. Each GV of a participant has the potential to pass on their genes, thus both generations (children and grandchildren) are tallied. Final analyses were conducted on number of living children, and GV as well as the number of living grandchildren and the number of living grandchildren controlling for number of living children (this will assess offspring’s rate of producing grand-offspring) as dependent variables. Infant mortality was calculated from the number of deceased children over the total number of children born to the participants. Height, weight, and HGS were also assessed. A Riester Dynatest Hand Dynamometer was used to record HGS. Handgrip strength was taken three times for the dominant hand and the single highest result was recorded in bars (the bar being a unit of pressure equal to slightly more than one unit of Earth’s atmospheric pressure at sea level). For voice samples, participants were asked to speak into a dictation microphone and count from one to 10 in their native language. Audio files were recorded with 11 kHz, 16 bit mono and were adequate for extracting pertinent acoustic parameters. Acoustic analyses were conducted using Praat software [86] and involved measuring fundamental frequency using Praat’s autocorrelation algorithm. Fundamental frequency was calculated for each vowel across counting utterances from one to ten (Imue, imbari, indatu, ine, indano, hamboumue, hambombari, hambondatu, muviu, omurongo). Mean fundamental frequency values were then calculated and used in subsequent analyses.

## Results

Descriptive statistics for age, GV, number of living children, grandchildren, height, weight, mean fundamental frequency, and HGS values are shown in Table 1. Hierarchical regression models were used to assess the effects of targeted predictor variables on various measures of reproductive success. Prior to analysis, data were checked for normality and the presence of both univariate and multivariate outliers. Three males were dropped from the analysis due to having one of their RS Z-scores above 3.33 (*p*<.001); 2 for having a large number of children and one for having both a large number of children and a large number of grandchildren. All analyses conducted on males were re-analyzed using log-transformed values which did not change any of the results. The analyses of male participants using log transformed variables appears in the supporting information section (Supporting Information S1 and Table S1). All individuals under the age of 18 were removed from statistical analyses. The final analyses were conducted on 54 females and 36 males. All analyses used one-tailed tests for significance due to *a priori* predictions; higher fundamental frequency in females and lower fundamental frequency in males was hypothesized to correlate with RS measures and high handgrip strength within each sex was hypothesized to correlate with RS measures. Only 5 individuals in the sample (3 females and 2 males) reported a death of one or more of their children, making a statistical analysis of both mortality rates and number of births [e.g., 26] not feasible. This was likely due to a reticence on the part of participants to discuss child mortality, as opposed to low rates of child mortality among participants [87].

**Figure 1:**
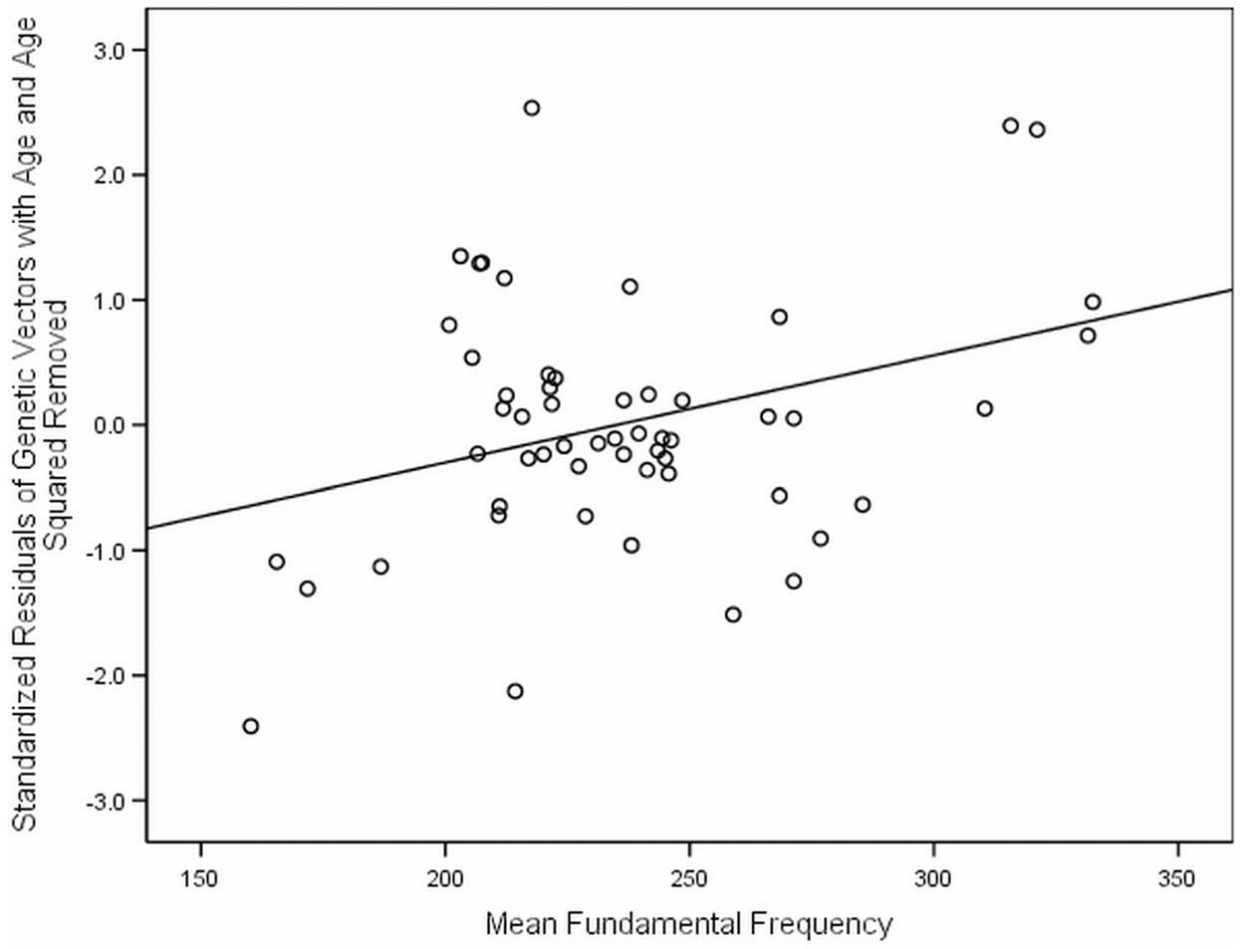
The relationship between Himba female genetic vectors and fundamental frequency. Residual values from regression of genetic vectors with age and age^2^ removed, plotted against differences in fundamental frequency. As fundamental frequency increased, so did Himba female genetic vectors, over and above age variables.

Similar to findings from Apicella et al. [26], there were linear and quadratic effects of age on RS variables (multiple R^2^ = .31; *p* = .001). Thus, age and age^2^ were entered into the first regression block for all analyses to control for their effects on RS variables prior to entering variables of interest. Height and weight were initially entered in the second block, but dropped from all subsequent models for females as they were non-significant predictors of RS variables after age and age^2^ had been incorporated (*p*>.30, whether entered individually or simultaneously). Fundamental frequency was then entered in the manner used by Apicella et al. [26]. Table 2 and 3 show all results for the hierarchical regression models for fundamental frequency in females and males predicting GV and number of living children. For females, fundamental frequency was a significant predictor of all RS variables (see Figure 1 and Table 4), including the number of living grandchildren (*β*.239; *p*<.05) and number of living grandchildren controlling for the number of living children (*β = *.168; *p* = .05). Full results revealing findings for height, weight, body mass index (BMI), and a discussion on controlling for these variables in relation to targeted predictor variables used in the present study can be found in the supporting information section (Supporting Information S1, Table S2 and Table S3).

**Table 2:** Hierarchical regression analyses: Variables predicting genetic vectors and number of children (in parentheses) for females (N = 54).

	model 1				model 2			model 3		
variable	*B*	*SE B*	*β*	*B*		*SE B*	*β*	*B*	*SE B*	*β*
age	.279	.119	1.602*	.298		.113	1.714**	.265	.110	1.52**
	(.311)	(.091)	(2.49)**	(.323)		(.089)	(2.58)**	(.295)	(.085)	(2.36)**
age^2^	−002	.001	−1.080	−.002		.001	−1.144*	−.002	.001	−.821
	(−.003)	(.001)	(−2.23)**	(-.003)		(.001)	(−2.28)**	(−.003)	(.001)	(−1.9)**
**fundamental frequency (Hz)**				.024		.009	**.285** **	.025	.009	**.292** **
				(.014)		(.007)	**(.236)** *	(.015)	(.007)	**(.245)** *
**handgrip strength (bars)**								.075	.032	**.281** *
								(.063)	(.025)	**(.327)** **

* *p*<.05.

** *p*<.01 all tests are one-tailed.

Age, age^2^ were entered as control variables. The effects of height and weight were also removed from handgrip strength before the analysis. Fundamental frequency and handgrip strength were entered as target variables (bold). Genetic vectors composes of the total number of surviving children plus the number of grandchildren (halved because of genetic relatedness), or (number of children + (.5×grandchildren)).

**Figure 2:**
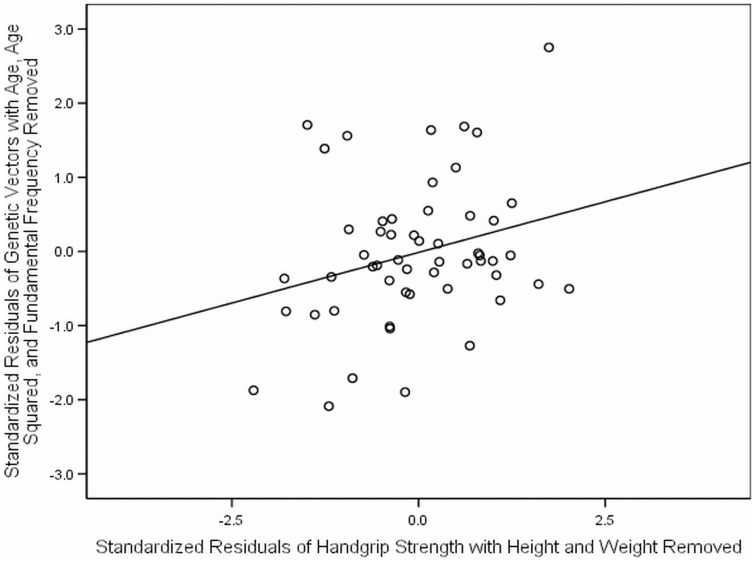
The relationship between Himba female genetic vectors and handgrip strength. Residual values from regression of genetic vectors with age, age^2^, and fundamental frequency removed, plotted against differences in handgrip strength residuals. As handgrip strength increased, so did Himba female genetic vectors, over and above the other predictor variables.

Because height and weight correlate with HGS [21], the next model assessed the impact of HGS by using residual values of HGS, with the effects of height and weight removed. Residualized-HGS (*r*-HGS) remained a significant predictor of GV over and above age, fundamental frequency and body dimensions (see Figure 2). Fundamental frequency and *r*-HGS together explained 13.9% of the variance in GV and 13.5% of the variance in number of living children among females. Running the same regression without fundamental frequency revealed that *r*-HGS was a significant predictor of GV (*β = *.236; *p* = .035) and number of living children (*β = *.277; *p* = .024) but not number of grandchildren (*β = −*.074; *p* = .233), nor number of grandchildren controlling for the number of children (*β = *−.030 *p* = .860) in females. Fundamental frequency was not correlated with *r*-HGS for either sex (females; *p* = .765; males; *p* = .157) showing independent contributions of each variable in predicting GV and number of living children. This same analysis was conducted among younger females (49 and under & 39 and under), and fundamental frequency and *r*-HGS were not correlated (see Supporting Information S1).

The same regression models applied to males revealed that neither fundamental frequency nor *r*-HGS predicted any of the four RS variables investigated (see Table 3 for GV and number of children results).

**Table 3:** Hierarchical regression analyses: Variables predicting genetic vectors and number of children (in parentheses) for males (N = 36).

	model 1				model 2				model 3		
variable	*B*	*SE B*	*β*	*B*		*SE B*	*β*	*B*		*SE B*	*β*
age	.606	.219	2.47**	.611		.222	2.489**	.654		.24	2.667**
	(.618)	(.178)	(3.18)**	(.627)		(.179)	(3.22)**	(.63)		(.194)	(3.23)**
age^2^	−005	.002	−1.936*	−.005		.002	−1.943*	−.005		.002	−2.047*
	(−006)	(.002)	(−2.77)**	(−.006)		(.002)	(−2.79)**	(−.006)		(.002)	(−2.79)**
**fundamental frequency (Hz)**				−.01		.028	**−.048**	.008		.029	**-.04**
				(−.018)		(.023)	**(-.115)**	(−.018)		(.023)	**(-.114)**
**handgrip strength (bars)**								.032		.061	**.107**
								(.003)		(.05)	**(.012)**

* *p*<.05.

** *p*<.01 all tests are one-tailed.

Age, age^2^ were entered as control variables. The effects of height and weight were also removed from handgrip strength before the analysis. Fundamental frequency and handgrip strength were entered as target variables (bold). Genetic vectors composes of the total number of surviving children plus the number of grandchildren (halved because of genetic relatedness), or (number of children + (.5×grandchildren)).

To investigate whether these effects were retained in older females to assess the Grandmother Hypothesis, two models were constructed with age cut-offs at 40 and 50 years. Residualized HGS was recalculated among each age group in the same manner as with the whole sample. Among the 27 Himba women over the age of 39, *r*-HGS (*β = *.477; *p* = .017) and fundamental frequency (*β = *.422; *p* = .017) were significant predictors of GV. Residualized HGS (*β = *.6; *p* = .002) and fundamental frequency (*β = *.324; *p* = .048) were also significant predictor variables when using number of living children as the dependent variable. Fundamental frequency was also a significant predictor of the number of living grandchildren (*β = *.346; *p* = .024) and number of living grandchildren controlling for the number of living children (*β = *.277; *p* = .047), but *r*-HGS was not a significant predictor of these RS variables. Among the 19 women over the age of 49, *r*-HGS (*β = *.439; *p* = .035) and fundamental frequency (*β = *.529; *p* = .01) were significant predictors of GV. Residualized HGS (*β = *.582; *p* = .008) and fundamental frequency (*β = *.388; *p* = .025) were also significant predictors of the number of living children. In addition, fundamental frequency was a large and significant predictor of the number of living grandchildren (*β = *.500; *p* = .007) and the number of living grandchildren controlling for the number of living children (*β = *0.425; *p* = .016).

Lastly, to examine the effects of *r*-HGS by itself for the 27 females over the age o 39, *r*-HGS was a significant predictor of living children and GV when entered into the regression model without fundamental frequency (number of living children; *β = *.613; *p* = .003, GV; *β = *.494; *p* = .015), but not for number of living grandchildren (*β = *.059; *p* = .074) nor number of living grandchildren controlling for number of living children (*β = * −.091; *p* = .634). For the 19 females over the age of 49, *r*-HGS was a significant predictor of the number of children (*β = *.599; *p* = .012), but not for GV (*β = *.461; *p* = .064), nor for number of grandchildren (*β = *.018; *p* = .467) nor of the number of grandchildren controlling for the number of children (*β = *.004; *p* = .987). All significant findings showing standardized regression coefficients for fundamental frequency and *r-*HGS predicting all four RS variables in females are summarized in Table 4.

**Table 4:** Summary of significant findings (*β* = standardized regression coefficients) for fundamental frequency and residualized handgrip strength predicting reproductive success variables when entered simultaneously into regression models.

variables	children (*β)*	grandchildren (*β)*	genetic vectors (*β)*	grandchildren (controlling for children)(*β)*
**all females (N = 56)**				
fundamental frequency	**.245** *	**.239** **	**.292** **	**.168** *
residualized handgrip strength	**.327** **	N.S.	**.281** *	N.S.
**females 39+(N = 27)**				
fundamental frequency	**.324** *	**.346** *	**.422** *	**.277** *
residualized handgrip strength	**.600** **	N.S.	**.477** *	N.S.
**females 49+(N = 19)**				
fundamental frequency	**.388** *	**.500** **	**.529** *	**.425** *
residualized handgrip strength	**.582** **	N.S.	**.439** **	N.S.

* *p*<.05.

** *p*<.01.

## Discussion

Female fundamental frequency and HGS were unique predictors of lifetime genetic fitness measures among indigenous Himba women. Females with higher-pitched voices and relatively stronger handgrip strengths tended to have higher reproductive outcomes. HGS was a robust predictor of both GV and number of living children, particularly among elderly females, which strongly suggests that HGS is a good measure of phenotypic quality and (Darwinian) successful ageing.

Past research has shown that females with a high voice pitch are rated as sounding younger, more attractive and more feminine [e.g., 6,10]. This trait has been proposed as a mate quality signal [5], [10], [19,] and is thought to be related to features of fitness. Among females, our results demonstrate a direct relationship between fundamental frequency and several measures of genetic fitness.

Although HGS is sexually dimorphic, it is important to emphasize that it remains an excellent indicator of physical and developmental health in both men and women [46], particularly in the elderly [49], [88] and was the largest predictor of the genetic vector variable and number of living children in this population. The Himba live under harsh environmental conditions where survival is dependent on the physical attributes of both sexes. However, increased female strength in this population does not necessarily imply increased masculinity (and thus decreased femininity), but rather signals overall vitality, health, and the ability to protect and provision offspring, particularly later in life. More importantly, these results provide evidence that the theoretically imposed dichotomy used in many indigenous mate preference studies, where a hypothetical male can either choose an attractive and feminine mate or a more masculine (and presumably “harder working”) mate, may be an overly simplified characterization of mate choice [e.g., 89] that neglects the role of phenotypic quality. In the present study, women with more feminine voices who were *also* stronger than would be expected, given their height, weight and age had more progeny. In essence, a Himba male could maximize his fitness by mating with a relatively strong and feminine mate. Our results are most parsimoniously explained by the wealth of evidence that links higher HGS to increased phenotypic quality in both sexes, particularly at later ages, as opposed to positing that Himba women with higher fitness have feminine voices but masculine bodies.

However, there were some limitations to HGS. It did not predict the number of grandchildren, with or without statistically controlling for the number of children, whereas fundamental frequency did, thus caution is warranted when interpreting the current results, particularly in relation to the Grandmother Hypothesis; These findings are not conclusive ‘proof’ of the Grandmother Hypothesis, but do provide evidence in support of the theory.

The current results suggest that assumption of sexually dimorphic traits only benefiting one sex may be unfounded. A premium has undoubtedly been placed on increased male strength during human evolution [e.g., 90,91], but having adequate levels of upper-arm or handgrip strength appears to be important for females as well, especially in indigenous environments that require both sexes to engage in strenuous manual labor for the majority of their lives. Among the Hadza, Hawkes et al. [80] found that among females, the hardest workers were grandmothers and great aunts who spent most of their day engaging in strenuous manual labor bringing home much needed food for their younger kin. Their efforts translated into faster growth rates of grandchildren [74] and increased survival rates during periods of food shortage [75]. Increased HGS would be vitally important in a Hadza female’s daily duties and could impact the quality and quantity of food gathered. Although the Himba are culturally distinct from the Hadza, they are undoubtedly united in their need to work hard to provide food for their family. Within this sample, stronger Himba mothers, particularly older mothers, had more children, possibly as a direct result of their strength.

Investigating evolutionary hypotheses among populations resembling those that prevailed during human evolutionary history may be more valid than comparable investigations in Western societies. This is important since the effects found in this study might be less pronounced in a modern population. Women from 1^st^ world nations are less likely to be engaged in strenuous physical activities (e.g., sports, manual labor), which could lead to null findings between RS variables and HGS. This pattern has been found for height in 1^st^ vs. 3^rd^ world populations, where the benefits of being tall (or taller than average) are generally found for 1^st^ world males and 3^rd^ world females only [92]–[96] but not vice versa.

While the phenotypic quality and acoustic parameters we examined correlated with several measures of RS, no effects were found among males. These sex differences could be due to several factors. The male sample was smaller and significantly older, and potentially subject to non-random sampling as many younger, presumably able-bodied males were often absent from villages during the day to herd [85], personal observations of P.S., A.S. and M.M.]. Paternal certainty is another concern, particularly among indigenous cultures like the Himba where extra-paternity rates are high [87]. Another limitation of our dataset would be the reliance on retrospective reports. Child mortality rates were lower than expected, and the transformation of age into a numerical Western representation might have affected our results. In conjunction with limited visitation time, it is possible that the family history data were not completely accurate and thus caution is warranted when interpreting these results.

Residualized HGS was a significant predictor of the number of living children and GV in both groups of older females but did not predict the number of living grandchildren. Fundamental frequency was a significant predictor of all reproductive variables in both groups of older females. Although both age-truncated analyses had lower sample sizes, the relationship between strength, fundamental frequency and measures of reproductive success might be expected to diminish in older females, as the onset of menopause is associated with lower levels of cyclic hormones necessary to maintain normal vocal function [19] and other feminine mate quality features linked to reproductive potential. Additionally, in spite of other physiological changes at menopause, a growing body of evidence suggests that strength is well preserved in post-menopausal females. Although some research has shown that muscle mass and strength decline with age [e.g., 97], this may be due to senescence and inactivity [98], [99], and may not be due to menopause per se. Indeed, other studies have shown that handgrip strength is unaffected by menopause [100] and that ovarian testosterone levels (but not circulating testosterone levels) actually increase as a function of age in menopausal females [101]. Although a sedentary lifestyle leads directly to a decline in strength [97], the effect is mitigated by resistance training [99], [102], a history of manual labor [98] as well as proper nutrition and adequate protein intake [99]; the very conditions under which the Himba live. This environment, although different in some ways, does resemble the human environment of evolutionary adaptedness (certainly more so than First World environments). As such, there is a high physical workload for all females which continues well after reproductive age. This would tend to buffer against decreases in strength, which are more common in sedentary First World populations. Thus, our data show that older women who are stronger have higher measures of genetic fitness. Strength could be one of the proximal contributors to the ability of post-menopausal women to augment the fitness of their children and grandchildren, which lends support to the Grandmother Hypothesis. Therefore, not only should the presence or absence of elderly women be considered when investigating the Grandmother Hypothesis, but the type of help that can be obtained and the ability of a grandmother to engage in this help should be incorporated as well.

In conclusion, our findings demonstrate for the first time a relationship between several measures of female genetic fitness and fundamental frequency among a group of indigenous-living females. We also show that HGS, a well documented marker of phenotypic quality explains a significant amount of variance in several measures of female genetic fitness, particularly among elderly females, which we argue provides support for the Grandmother Hypothesis.

## Supporting Information

Table S1 **Z-scores for height, weight, fundamental frequency, HGS and** ***r*** **-HGS for the 3 outlier males and mahalanobis distance (D) for these variables.** The critical value for D at k = 5 is 20.52 (*p* = .001).
(DOC)

Table S2 **Hierarchical regression analyses: Variables predicting genetic vectors and number of living children (in parentheses) for males (N = 36).** Age and age^2^ were entered as control variables. Height and weight were entered as target variables.
(DOC)

Table S3 **Hierarchical regression analyses: Variables predicting genetic vectors and number of living children (in parentheses) for females (N = 54).** Age and age^2^ were entered as control variables. Height and weight were entered as target variables.
(DOC)

Supporting Information S1 **Supplemental results include analyses of correlations between fundamental frequency and r-HGS in pre-menopausal women, z-scores and mahalanobis distance for acoustic and physical variables for male outliers, retaining male outliers using log-transformed variables, the effects of height and weight on RS variables for males and females, and a discussion of height and weight in the context of the present data.**
(DOC)
