# Supplementary material for: Voice and Handgrip Strength Predict Reproductive Success in a Group of Indigenous African Females
Source: PLoS One. 2012 Aug 3;7(8):e41811. doi: 10.1371/journal.pone.0041811 (PMC3411669; doi:10.1371/journal.pone.0041811)
Supplement: Table S1 — Z-scores for height, weight, fundamental frequency, HGS and r -HGS for the 3 outlier males and mahalanobis distance (D) for these variables. The critical value for D at k = 5 is 20.52 (p = .001). (DOC) [file pone.0041811.s001.doc]

|  | height | weight | fundamental frequency | HGS | *r-*HGS | mahalanobis distance for variables to the left |
| --- | --- | --- | --- | --- | --- | --- |
| outlier #1 | 0.28563 | 0.37569 | -1.09797 | -1.23084 | -1.29356 | 4.56810 |
| outlier #2 | 1.12109 | 0.37569 | 0.07917 | -0.71364 | 0.21822 | 4.27080 |
| outlier #3 | 1.12109 | 1.42224 | 0.06049 | -1.10154 | -0.96274 | 4.01418 |
