# Supplementary material for: Voice and Handgrip Strength Predict Reproductive Success in a Group of Indigenous African Females
Source: PLoS One. 2012 Aug 3;7(8):e41811. doi: 10.1371/journal.pone.0041811 (PMC3411669; doi:10.1371/journal.pone.0041811)
Supplement: Table S2 — Hierarchical regression analyses: Variables predicting genetic vectors and number of living children (in parentheses) for males (N = 36). Age and age2 were entered as control variables. Height and weight were entered as target variables. (DOC) [file pone.0041811.s002.doc]

|  | model 1 | | | model 2 | | | model 3 | | |
| --- | --- | --- | --- | --- | --- | --- | --- | --- | --- |
| variable | *B* | *SE B* | *Β* | *B* | *SE B* | *β* | *B* | *SE B* | *β* |
| age | .606  (.618) | .219  (.178) | 2.47**  (3.17)** | .581  (.605) | .223  (.182) | 2.36**  (3.10**) | .451  (.47) | .223  (.175) | 1.837*  (2.42*) |
| age^2^ | -.005  (-.006) | .002  (.002) | -1.821*  (-2.77*) | -.003  (-.005) | .002  (.002) | -1.821  (-2.7**) | -.003  (-.004) | .002  (.002) | -1.270  (-1.97*) |
| **height (m)** |  |  |  | .067  (.036) | .085  (.070) | **.109**  **(.073)** | .016  (-.17) | .086  (.067) | **.026**  **(-.035)** |
| **weight (kg)** |  |  |  |  |  |  | .133  (.138) | .067  (.053) | **.289***  **(.376**)** |

**p* < .05, ***p* < .01 all tests are one-tailed
