# Supplementary material for: Voice and Handgrip Strength Predict Reproductive Success in a Group of Indigenous African Females
Source: PLoS One. 2012 Aug 3;7(8):e41811. doi: 10.1371/journal.pone.0041811 (PMC3411669; doi:10.1371/journal.pone.0041811)
Supplement: Table S3 — Hierarchical regression analyses: Variables predicting genetic vectors and number of living children (in parentheses) for females (N = 54). Age and age2 were entered as control variables. Height and weight were entered as target variables. (DOC) [file pone.0041811.s003.doc]

|  | model 1 | | | model 2 | | | model 3 | | |
| --- | --- | --- | --- | --- | --- | --- | --- | --- | --- |
| variable | *B* | *SE B* | *β* | *B* | *SE B* | *β* | *B* | *SE B* | *β* |
| age | .275  (.308) | .119  (.092) | 1.588*  (2.45**) | .272  (.306) | .120  (.093) | 1.570*  (2.44**) | .275  (.308) | .121  (.094) | 1.584  (2.45**) |
| age^2^ | -.002  (-.003) | .001  (.001) | -1.077  (-2.2**) | -.002  (-.003) | .001  (.001) | -1.049  (-2.18**) | -.002  (-.003) | .001  (.001) | -1.063  (-2.2**) |
| **height (m)** |  |  |  | .045  (.023) | .073  (.056) | **.072**  **(.052)** | .017  (.003) | .085  (.066) | **.028**  **(.006)** |
| **weight (kg)** |  |  |  |  |  |  | .026  (.020) | .042  (.033) | **.084**  **(.088)** |

**p* < .05, ***p* < .01 all tests are one-tailed
