# Supplementary material for: Voice and Handgrip Strength Predict Reproductive Success in a Group of Indigenous African Females
Source: PLoS One. 2012 Aug 3;7(8):e41811. doi: 10.1371/journal.pone.0041811 (PMC3411669; doi:10.1371/journal.pone.0041811)
Supplement: Supporting Information S1 — Supplemental results include analyses of correlations between fundamental frequency and r-HGS in pre-menopausal women, z-scores and mahalanobis distance for acoustic and physical variables for male outliers, retaining male outliers using log-transformed variables, the effects of height and weight on RS variables for males and females, and a discussion of height and weight in the context of the present data. (DOC) [file pone.0041811.s004.doc]

**Results for premenopausal females**

Females aged 39 and under as well as 49 and under were examined separately to determine if there was a correlation between HGS (also *r-*HGS) and fundamental frequency. In theory, post-menopausal women could be occluding a significant correlation between these variables due to the hormonal changes that occur at menopause. In other words, although there was no correlation for the sample as a whole, it is possible that for the subset of premenopausal women there could indeed be a significant correlation between HGS and fundamental frequency. For the 35 females aged 49 and under, there were no significant correlations (*r-*HGS: *p* =.621, *r* =-.087; HGS *p* =.808, *r* =.043). For the 28 females aged 39 and under there were also no significant correlations (*r*-HGS: *p* =.614, *r* =-.102; HGS: *p* =.440, *r* =.155).

**Results for reproductive outlier males on fundamental frequency and handgrip strength.**

The 3 males who were excluded from primary analyses had the z-scores for height, weight, HGS, *r*-HGS and fundamental frequency analyzed. These results appear in Table S1. There were no z-scores larger than 1.2 in absolute magnitude. Additionally, all physical variables (HGS, *r*-HGS, fundamental frequency, height and weight) were entered simultaneously for all male subjects to determine if any male participant was a multivariate outlier, as assessed by mahalanobis distances. None of the 3 outliers were multivariate outliers, nor was any male in the dataset as a whole.

**Results for outlier males by log-transformation of reproductive success variables.**

Including the three males who were removed from primary analyses due to high numbers of progeny by log-transforming the variables (children, grandchildren and genetic vectors (GV)) did not change any results. In other words, there were no new significant findings. When entered into a hierarchical regression along with age, age^2^, height and weight, *r-*HGS did not predict log-transformed number of children (*β* =-.102, *p* = .463), log-transformed grandchildren (*β* =-.437, *p* = .087) or log-transformed GV (*β* =-.104, *p* = .456). Likewise, when entered after the above-mentioned control variables, fundamental frequency did not predict log-transformed number of children (*β* =-.032, *p* = .844), log-transformed grandchildren (*β* =-.276, *p* = .185) or log-transformed GV (*β* =-.162, *p* = .157).

**Results for height and weight;**

Full results for the regression models for height and weight can be found in Table S2 (for males) and Table S3 (for females). Male weight was a significant predictor for two reproductive variables (children and GV) but not grandchildren. Height was not a significant predictor for either sex. Additionally, among males, BMI was also a significant predictor on its own for number of living children (*β* =.302, *p* = .015) but not for GV (*β* =.217, *p* = .123). When entered with or without height, both BMI and weight were non-significant, indicating shared variance between weight and BMI.

**Height and weight discussion:**

In this sample there was no association between reproductive variables and height or weight in women, and only an association between weight (and BMI) in males. Height and weight are both sexually dimorphic traits, and many first-world studies have determined that taller men and shorter women are considered more desirable as mates and have greater reproductive success (RS), with a caveat being that very short women are often found to have decreased RS [1,2,3]. However, several studies have found that in stressed populations (i.e., indigenous or Third World), taller women have higher RS [4,5]. Additionally, many studies have failed to uncover significant correlations in one or both sexes, or have even found negative relationships with either height and/or weight. For example, Sear [6] found no relationship between both height and weight and number of living children (to age 14) in rural Gambian males, although BMI was a significant predictor. While among both a !Kung population and a rural Bantu-speaking population in Namibia (the Kavango), taller men had higher reproductive success, yet among *urban* Namibian Bantu-speakers, it was the shorter men that had increased RS [6,7,8]. Among females in the same Gambian population mentioned above, Sear, Allal and Mace [4] did find a significant correlation between height and RS, but not with weight.

In conclusion, height and weight are predictors of RS in some instances for some populations. Some of the null findings for height and weight in the present study might be due to the smaller sample size and potential selection bias in the male sample. Lastly, it is important to note that due to paternity uncertainty, which is of particular concern among the Himba [9], all significant results in males must be interpreted with caution.

**References:**

1. Nettle D (2002) Women’s height, reproductive success and the evolution of sexual dimorphism in modern humans. Proc R Soc Lond B 269: 1919–1923
2. Nettle D (2002) Height and reproductive success in a cohort of British men. Hum Nat 13: 473-491.
3. Pawlowski B, Dunbar RI, Lipowicz A (2000) Tall men have more reproductive success. Nature 403: 156.
4. Sear R, Allal N, Mace R (2004) Height, marriage and reproductive success in Gambian women. Res Econ Anth 23: 203-224.
5. Monden CWS, Smitts J (2009) Maternal height and child mortality in 42 developing counries. Am J Hum Bio 21: 305-311.
6. Sear R (2006) Size-dependent reproductive success in Gambian men: Does height or weight matter more? Soc Bio 53: 172-188.
7. Kirchengast S (2000) Differential reproductive success and body size in !Kung San people from northern Namibia. Collegium Anthropol 24: 121-132.
8. Kirchengast S, Winkler EM (1995) Differential reproductive success and body dimensions in Kavango males from urban and rural areas in Northern Namibia. Hum Bio 67: 291-309.
9. Scleza BA (2011) Female choice and extra-pair paternity in a traditional human population. Biol Lett 7: 889-91.
